# The effects of synthetic estrogen exposure on premating and postmating episodes of selection in sex-role-reversed Gulf pipefish

**DOI:** 10.1111/eva.12093

**Published:** 2013-08-02

**Authors:** Emily Rose, Kimberly A Paczolt, Adam G Jones

**Affiliations:** 1 Department of Biology, Texas A&M UniversityCollege Station, TX, USA; 2 Department of Biology, University of MarylandCollege Park, MD, USA

**Keywords:** EE2, Gulf pipefish, selection, sex-role-reversed mating system, synthetic estrogen

## Abstract

Environmental estrogens have been shown to affect populations of aquatic organisms in devastating ways, including feminization of males, alterations in mating behaviors, and disruption of sexual selection. Studies have shown 17α-ethinylestradiol (EE2) exposure to induce female-like secondary sexual traits in male Gulf pipefish, changing how females perceive affected males. We aimed to understand the effects of EE2 exposure on the sex-role-reversed mating system and the strength of selection in Gulf pipefish. We used artificial Gulf pipefish breeding aggregations and microsatellite-based parentage analysis to determine maternity. We then calculated the opportunity for selection and selection differentials on body size for both sexes during three consecutive episodes of selection. Exposure to EE2 did not affect the strength of selection, likely due to the unusual sex-role-reversed mating system found in this species. With respect to multiply mated females, EE2-exposed females produced more eggs with higher embryo survivorship than nonexposed females. Thus, short-term exposure to low concentrations (2.0 ng/L) of EE2 in Gulf pipefish enhanced female reproductive success. However, higher EE2 concentrations (5.0 ng/L) caused complete reproductive failure in Gulf pipefish males. These results call for more work on the long-term effects of EE2 exposure in Gulf pipefish in artificial and natural populations.

## Introduction

Endocrine disrupting chemicals can mimic natural hormones and alter bodily processes regulated by the endocrine system, causing detrimental effects on reproduction and hormone production (Orlando and Guillette 2007). The earliest studies on the impacts of endocrine disruptors focused primarily on the negative physiological effects of these compounds on the reproductive systems of exposed animals, particularly in aquatic organisms such as frogs and fish (Allen et al. 1999; Iguchi et al. 2001). These studies documented partial feminization or complete sex reversal in exposed males and termination of egg production in females (Länge et al. 2001; Islinger et al. 2003; Xu et al. 2008). One endocrine disruptor that has received recent attention is a synthetic estrogen found in hormonal contraceptives called 17α-ethinylestradiol, also known as EE2. This contaminant is resistant to degradation in the body and ultimately ends up in the aquatic environment by passing through domestic wastewater treatment facilities and being released as a biologically active molecule in the effluent where it can accumulate to levels that cause deleterious effects on exposed organisms (Kolpin et al. 2002). For instance, EE2 has been detected in US rivers at levels as high as 820 ng/L, European locations at 35 ng/L, and at 42 ng/L in Canadian sewage treatment effluent (Ternes et al. 1999; Kolpin et al. 2002; Pojana et al. 2007). These high contamination levels raise questions regarding the types of effects that varying levels of EE2 exposure could have on populations of economically or ecologically important species that occupy such sites during all or part of their life cycles (Segner et al. 2003; Soares et al. 2009).

Lower ranges of EE2 have been detected in surface waters near sewage treatment plants, generally ranging from below 0.5 to 5 ng/L in locations worldwide (Allen et al. 1999; Ternes et al. 1999; Johnson et al. 2000). Chronic exposure to lower concentrations of EE2 has been shown to cause entire populations of fathead minnow (*Pimephales promelas*) to stop reproducing after a single generation of 5–6 ng/L exposure (Kidd et al. 2007). Relatively low levels of EE2, ranging from 0.5 to 5.0 ng/L, have been shown to have drastic effects on gene expression levels, reproductive development, and behavior in exposed populations (Nash et al. 2004; Larsen et al. 2008; Ferreira et al. 2009; Soares et al. 2009). Even if endocrine disruptors have no obvious morphological or physiological effects, changes in either gene expression levels or behavior may hinder the organism's reproductive success.

Exposure to endocrine disruptors, such as EE2, can alter an organism's mating behaviors at several different levels of biological organization. For example, endocrine disruptors can improperly induce or eliminate secondary sex traits, decrease sexual behaviors such as courtship displays or male territory aggression, disrupt communication between the sexes, or simply sterilize the exposed animals by interfering with the normal development of reproductive structures (Orlando and Guillette 2007; Coe et al. 2008; Munakata and Kobayashi 2009; Saaristo et al. 2009b, 2010). In medaka fish (*Oryzias latipes*), for instance, EE2 has been shown to disrupt mating rituals, such as dancing and following with their mates; however, normal behaviors such as resting and swimming were not affected (Oshima et al. 2003). Endocrine disruptors have been shown to alter courtship displays and aggression in several other species of fish including goldfish (*Carassius auratus*), zebrafish (*Danio rerio*), three-spined sticklebacks (*Gasterosteus aculeatus*), salmon (*Oncorhynchus masou)*, and guppies (*Poecilia reticulata*) (Bell 2001; Bjerselius et al. 2001; Kristensen et al. 2005; Colman et al. 2009; Sarria et al. 2011a). Several of these studies demonstrated drastic changes in male behaviors, such as males completely failing to court females or no longer defending their territories, at EE2 concentrations as low as 0.5–3 ng/L (Bjerselius et al. 2001; Oshima et al. 2003; Colman et al. 2009). Sarristo et al. found that EE2 exposure affects courtship, aggression, and parental care in male sand gobies, *Pomatoschistus minutus* (Saaristo et al. 2009a, 2010). Additionally, male sand gobies exposed to EE2 experienced difficulty defending their nests, causing females to prefer nonexposed males when given the choice (Saaristo et al. 2009b). This study demonstrates the strong impact EE2 can have on an exposed male's mating success by disrupting the mating system and decreasing the importance of secondary sexual characteristics in males.

We chose to study the effects of EE2 on sexual selection in the sex-role-reversed Gulf pipefish, *Syngnathus scovelli,* because this species resides in areas that are likely to be affected by EE2 and has previously been the focus of many behavioral studies. Gulf pipefish are found in seagrass beds along the Gulf of Mexico and Atlantic coastlines of North America (Dawson 1982) and depend on the seagrass community for their habitat, food, and protection. This reliance on the seagrass ecosystem ties the organisms to locations near the coast, often in the vicinity of sewage treatment plants and other sources of environmental contamination. There are numerous benefits to using the Gulf pipefish mating system to test the effects of EE2 on the strength of sexual selection. The Gulf pipefish, like the other members of the family Syngnathidae, exhibits an evolutionarily novel trait in male pregnancy and as a result is sex-role reversed, meaning that females compete for access to mates and males act as the choosy sex during courtship. Females of this species are typically larger than males at sexual maturity, and males prefer larger females (Jones and Avise 2001). Gulf pipefish are sexually dimorphic with respect to secondary sexual traits as well, with sexually mature females exhibiting iridescent bands on their trunk, a deeply keeled abdomen, and an enlarged, darkened dorsal fin (Dawson 1982). None of these traits normally occur in males. The Gulf pipefish has a polyandrous mating system, where males typically mate with a single female per pregnancy but successful females transfer eggs to multiple mates (Jones and Avise 1997). Consequently, sexual selection acts more strongly on females (Jones et al. 2001). Pregnant male Gulf pipefish carry eggs in a sealed brood pouch, located on the ventral surface of his body, where the eggs that have been transferred from the female are encased and fertilized, resulting in assured paternity of the offspring (Jones et al. 2001). Unsuccessful eggs, or those that do not develop, persist within the brood pouch until the end of the pregnancy, making it easy to accurately count the number of successful and unsuccessful eggs transferred (Paczolt and Jones 2010).

Our work builds on several studies that have addressed the effects of endocrine disruptors on various species of pipefish (Ripley and Foran 2008; Sarria et al. 2011b). For example, Sarria et al. (2011c) found that the juvenile black striped pipefish, *S. abaster*, when exposed to both tributyltin (TBT) and EE2, altered their swimming patterns and their response to the mosquitofish (*Gambusia affinis*), a potential predator. The Gulf pipefish has recently emerged as a useful model for testing the effects of endocrine disruptors. For instance, EE2 levels of 1 and 100 ng/L have been shown to affect male morphology, gene expression levels, and mating dynamics in the Gulf pipefish (Ueda et al. 2005; Partridge et al. 2010). As a result of EE2 exposure, male pipefish experience feminization by developing a deeper abdomen and iridescent bars, traits typically only seen in females. Female Gulf pipefish prefer nonexposed over exposed males, and exposed males were less likely to become impregnated when chosen as a mate (Partridge et al. 2010). These results from binary mate choice assays indicate that mating dynamics in Gulf pipefish are affected by EE2 exposure. Sex-role-reversed pipefish have also been shown to have high levels of natural estrogen in brooding males, a reversal of the normal pattern of estrogen in male teleosts, suggesting exposure to a synthetic estrogen, EE2, might affect selection acting on male pregnancy (Mayer et al. 1993). The next question, which we address here, concerns how this disruption in mating preferences alters the mating system and the strength of selection in pipefish breeding aggregations. We set out to determine the effects of low levels of EE2 exposure on both pre- and postmating episodes of selection in artificial breeding colonies of Gulf pipefish in a laboratory setting. To accomplish this goal, we measured mating success, reproductive success, and embryo survivorship within small breeding aggregations of Gulf pipefish, which were either exposed or not exposed to low concentrations of EE2 and used these data to measure the effects of EE2 on the intensity of selection and other reproductive attributes of this sex-role-reversed species.

## Methods

Gulf pipefish were collected from coastal seagrass beds near Aransas Pass, Texas (27°53′39.07″N, 97°7′51.69″W) from July through October 2010. Sexually mature males and females were separated by sex, acclimated to 26-ppt salinity tanks, and group housed in a flow-through system at Texas A&M University. All males were collected pregnant to confirm a history of successful reproduction and were allowed to give birth in the laboratory. Males were used in the experiment within a month of their collection to ensure a recent pregnancy. The EE2 concentrations at the collecting site are currently unknown. However, the location was chosen because of its long distance from any outflows from sewage treatment plants.

To determine the levels of EE2 exposure for our experiment, we performed a pilot study at EE2 concentrations of 2 and 5 ng/L with a total of eight males and eight females per treatment along with a parallel control set of fish. We placed males into the experiment while still pregnant with their broods from the field and monitored their abilities to carry their broods to term and to become pregnant with subsequent broods. While control males gave birth and mated soon thereafter as anticipated, several males exposed to 5 ng/L of EE2 experienced difficulties in giving birth and had dead offspring in their pouches after the first few days of exposure. None of the males exposed to 5 ng/L of EE2 had a second pregnancy in the laboratory and they appeared to resorb their brood pouches, apparently terminating their reproductive activities. Females, in contrast, continued to show courtship displays, including dancing and twitching, similar to the control females. Thus, we concluded from this pilot study that a concentration of 5 ng/L of EE2 would result in complete reproductive failure for Gulf pipefish, apparently mediated by a loss of reproductive ability for males and chose 2 ng/L as our EE2 concentration to allow for more typical pipefish mating and offspring development.

Over the course of the experiment, we conducted seven experimental replicates and seven control replicates. Each replicate began with eight nonpregnant, adult males and eight adult females in a 100-liter tank. For the experimental tanks, we used a 2 ng/L EE2 concentration, whereas the control tanks were EE2 free. The 17α-ethinylestradiol powder, of 98% purity, was obtained from Sigma (St. Louis, MO, USA; # 028K1411, MW 296.4, CAS 57-63-6) and dissolved in ethanol. Tanks treated with EE2 were initially dosed on the first day to obtain a concentration of 2 ng/L, and 10% water changes were conducted daily to maintain a constant level of 2 ng/L exposure as established by Partridge et al. (2010). Control tanks were treated with the same volume of ethanol (100 μL) without 17α-ethinylestradiol. We used 100-liter tanks that were optimized for length (92 × 30 cm), rather than height (38 cm), and were taller than previous tanks (28 cm) used for mating trials in Gulf pipefish (Paczolt and Jones 2010) to allow a large amount of surface area for fish matings while minimizing the amount of EE2 wastewater.

On the first day of the experiment, fish were marked with three visible implant fluorescent elastomer tags (VIFE; Northwest Marine Technology, Inc., Shaw Island, WA, USA) for identification using the protocol of Wood and Martin-Smith (2004). After anesthetization with diluted clove oil, each fish was marked on its tail with at least one blue and one yellow band to minimize color differences among marks. The marking procedure produced no mortalities, and we found no evidence for preferences for a single marking pattern during the experiment across both treatments (C: *F*_7,54_ = 0.911, *P *=* *0.5062; E: *F*_7,55_ = 1.369, *P *=* *0.2401). Fish were then randomly placed into treatments and were not significantly different in size across the two treatments (*t*-tests, males: *n* = 109, *P* = 0.6781; females: *n* = 111, *p* = 0.0904).

For 3 days before the establishment of mixed-sex breeding aggregations in the 100-L tanks, we housed males and females separately from one another and exposed them to the desired level of EE2 (i.e., 2 ng/L for experimental animals and 0 ng/L for control animals). On the fourth day of the experiment, sexes were combined in 100-liter tanks and the EE2 treatments were maintained throughout the rest of the experiment. Each replicate with eight males and eight females per tank was given 2 weeks during which mating took place. Males were checked daily for pregnancies and sacrificed using MS222 on day eight of their pregnancy. Eggs were dissected out of the male's pouch to determine the number of developing offspring and failed eggs. The proportion of normally developing embryos in each brood is used as a measure of embryo survivorship. Four offspring were removed from the top and bottom of the pouch and preserved in ethanol to use for assigning maternity. All nonpregnant males and females were sacrificed on day 18 of the experiment. Dorsal fins from the adult fish were preserved in ethanol for DNA extractions using the Genomic DNA Purification Kit from Gentra systems (Qiagen, Germantown, MD, USA). DNA was extracted from the embryos using a Chelex/proteinase K (20 mg/mL) extraction method.

We conducted a microsatellite-based parentage analysis to determine maternity of the broods using three highly variable microsatellites (*micro25.10*, *micro25.22*, and *micro22.3*) previously developed by for *S. scovelli* (Jones and Avise 1997). Paternity was already known because the offspring were dissected out of the male's brood pouch, where fertilization occurs and males are assured paternity (Jones and Avise 2001). Microsatellites were amplified for all adults and eight offspring per male with the exception of the eggs from males with zero offspring survivorship, and PCR products were sent for fragment analysis at the Cornell Life Sciences Core Laboratories Center. Microsatellite fragment sizes were measured by an Applied BioSystems 3730xl DNA Analyzer and analyzed using Peak Scanner software (Life Technologies, Grand Island, NY, USA). For each male's brood, a maximum of four alleles were represented, two of which matched the paternal genotype and two that represented the maternal genotype. Within each replicate, one of the eight females from the appropriate replicate was unambiguously matched with each set of embryos by exclusion. Maternity was easily assigned for all male broods, except for two males in the control tanks and one in the EE2 whose embryos failed to develop and thus were not amenable to microsatellite analysis. These three failed broods were excluded from further analysis.

Fish were photographed on the first, eighth, and fourteenth days of the experiment to measure standard body length and depth using ImageJ (NIH, Bethesda, MD, USA). Standard body length was measured from the snout of the fish to the end of the caudal peduncle, and standard depth was measured from the anterior end of the dorsal fin to the base of the fish's ventral surface. We calculated the covariance between standard length and relative fitness to measure the absolute selection differential, *s* (reported in cm), and standardized selection differential, *s*' (reported in units of phenotypic standard deviations; Lande and Arnold 1983). Absolute and standardized selection differentials were calculated across three episodes of sexual selection, broken down into one premating episode pertaining to mating success and two postmating episodes, including eggs transferred per mate and embryo survivorship (Arnold and Wade 1984a). These latter two episodes could be considered either sexual or natural selection, depending on the mechanisms involved. We also decomposed the variance in relative fitness, also known as the opportunity for selection (*I*), into components arising from these three episodes of selection (Arnold and Wade 1984a,b). We calculated *s*, *s*', and *I* for each tank individually and reported the means and standard errors for each episode across replicates. Relative fitness for each episode of selection was calculated separately for each replicate tank by dividing each measure of absolute fitness (i.e., number of mates, number of eggs transferred, or number of surviving offspring) by the corresponding mean absolute fitness across all the individuals of the same sex in the replicate. A more detailed analysis of the control tanks, including a more extensive discussion of the interpretation of the various phases of selection, has been published elsewhere (Rose et al. 2013). All other statistical tests were conducted using JMP 9.0 (SAS, Cary, NY, USA).

## Results

### Did EE2 have an effect on mating success?

**Figure 1:**
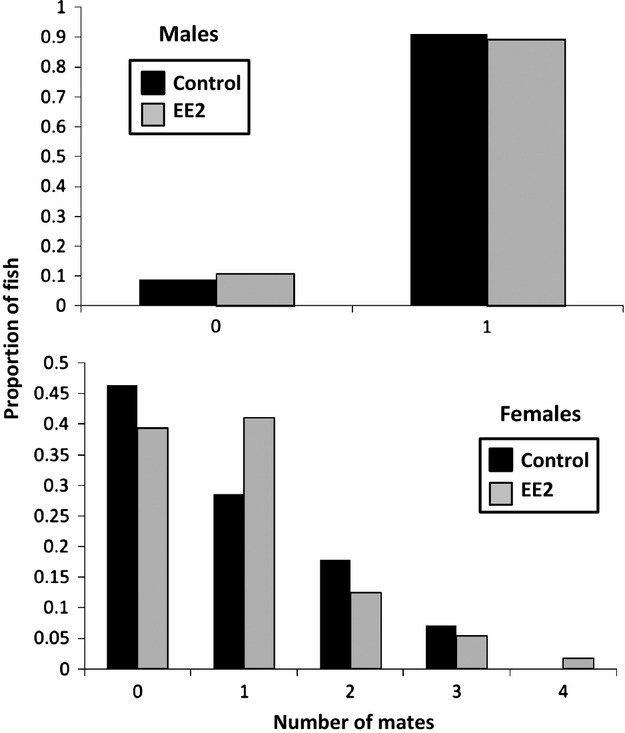
Histograms displaying mating success for males and females across both treatments with black bars for the control and gray bars for the EE2 treatment. The *y*-axis represents the frequencies, and the *x*-axis represents the number of mates.

The presence of low levels of EE2 did not have an effect on the number of males that mated successfully. Each treatment saw a similar number of males become pregnant: 91% of males mated in the control tanks with only five of the 56 males failing to become pregnant and 89% of the males mated in the EE2 treatment, where only six of 56 males remained unmated (Fig. 1). Parentage analysis of eight eggs per male, four from each end of the brood pouch, confirmed that all males mated with a single female, regardless of treatment. Using an anova with tank as a random effect, we found no effect of treatment on male size. However, despite a small sample size of unmated males, we did see a significant difference in size of mated and unmated males across the entire experiment (treatment: *F*_1,109_ = 0.005, *P *=* *0.945; mating category: *F*_1,109_ = 4.06, *P *=* *0.047; treatment*mating category: *F*_1,109_ = 0.074, *P *=* *0.787). Mated males averaged 8.73 cm (SE: 0.15) in length, while unmated males were on average 8.28 cm (SE: 0.25) in length.

The number of mated females was slightly higher in the EE2 treatment than that in the control, with 61% of females mating successfully (34 of 56) in the EE2 tanks as compared to 53% (29 of 55) in the control (Fig. 1). The control had more multiply mated females (*n* = 14) than the EE2 replicates (*n* = 11), whereas the EE2 treatment had more singly mated females (*n* = 23) than the control replicates (*n* = 15). Using an anova with replicate as a random effect, we found no significant effect of treatment and whether or not the female mated on female body length (ancova: treatment: *F*_1,110_ = 1.43, *P* = 0.254; female mating category: *F*_1,110_ = 3.225, *P* = 0.074; treatment*female mating category *F*_1,110_ = 0.448, *P* = 0.505).

### Did EE2 have an effect on reproductive success?

**Figure 2:**
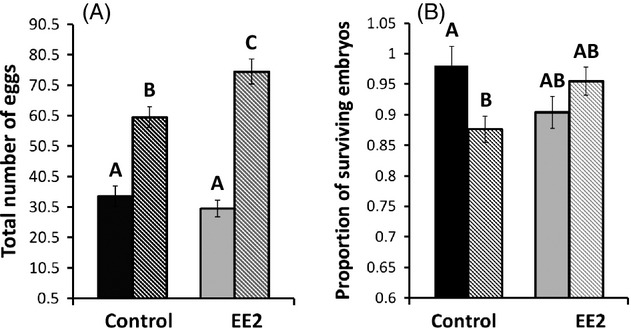
Number of eggs transferred and proportion of surviving embryos for females in the EE2 and control treatments. The graph on the left (A) shows the number of eggs females transferred to their mates over the entire experiment. Solid bars represent singly mated females, and striped bars represent multiply mated females. The graph on the right (B) shows the proportion of surviving embryos for singly mated (solid bars) and multiply mated (striped bars) males in the control and EE2 treatments. Within each figure, bars with shared letters are not significantly different from one another (Tukey's *post hoc* test). Error bars represent one standard error from the mean.

To examine female reproductive success, we compared the number of eggs females transferred throughout the whole experiment as a function of treatment and whether the female was unmated, singly mated, or multiply mated. We also used female body length as a covariate, included replicate as a random effect, and retained only the significant interactions in the final model (ancova: treatment: *F*_1,11_ = 2.17, *P *=* *0.164; female mating category: *F*_2,110_ = 246.5, *P *<* *0.0001; female body length: *F*_1,110_ = 3.61, *P *=* *0.06; treatment*females mating category: *F*_2,110_ = 4.48, *P *=* *0.014). As expected, multiply mated females transferred a greater number of eggs to males over the course of the experiment simply because they had gained access to more total brood pouch space (female mating category: *F*_2,110_ = 246.5, *P *<* *0.0001). On average across the treatments, multiply mated females transferred a total of 69 eggs compared with 32 eggs transferred per singly mated female. We also found a significant interaction between treatment and female mating category (treatment*female mating category: *F*_2,110_ = 4.48, *P *=* *0.014). This interaction was driven by the multiply mating females in the EE2 experiment transferring a significantly larger number of eggs than any other category of female, including multiply mating females in the control tanks (Fig. 2A). Thus, we did not see a difference between treatments in terms of the numbers of eggs transferred by singly mated females (Tukey's *post hoc*: *n* = 38, *P *=* *0.91), but we did observe that multiply mated females in the EE2 treatment transferred 15 more total eggs on average compared with multiply mated control females (Tukey's *post hoc*: *n* = 25, *P *=* *0.05).

We compared reproductive success on a per-brood basis across treatments and female mating categories, including singly or multiply mated. We used an ancova to examine the effects of treatment and female mating category on the number of surviving offspring with the total number of eggs initially transferred as a covariate. We included replicate as a random effect and reported only the significant interactions. In the control, females mating with multiple males experienced lower survivorship per embryo relative to singly mated females, whereas in the EE2 treatment, no such pattern was evident (ancova: treatment: *F*_1,96_ = 0.029, *P *=* *0.87; female mating category: *F*_1,96_ = 0.774, *P *=* *0.38; total eggs transferred: *F*_1,96_ = 1488, *P *<* *0.0001; treatment*females mating category: *F*_1,96_ = 7.66, *P *=* *0.007). Thus, in control tanks, females appear to experience a trade-off between number of eggs transferred and embryo survivorship, but this trade-off disappears in the EE2 treatment, despite the fact that multiply mating EE2 females actually transferred a greater number of eggs than multiply mating control females (Fig. 2A,B). This pattern is also evident from an embryo survivorship standpoint (Fig. 2B): an anova shows a significant difference in embryo survivorship across the experiment (*F*_3,95_ = 3.59, *P *=* *0.017).This pattern is a result of a significant difference between embryo survivorship for males mated with singly versus multiply mated females in control tanks (Tukey's *post hoc*: *n* = 47, *P *=* *0.033), but not between males mated with singly and multiply mated females in EE2 tanks (Tukey's *post hoc*: *n* = 50, *P *=* *0.446).

### Did EE2 have an effect on selection?

The decomposition of selection differentials is presented in Table 1. For both treatments, the largest contribution to the total selection on body size in females was from the first episode of selection, mating success. Selection on body size resulting from mating success in the EE2 females was significantly positive at *α *= 0.05, and we observed a similar, nonsignificant trend in control females. The selection differentials also provide evidence of a trade-off between pre- and postmating episodes of selection in control females, a result that was also apparent (and statistically significant) from our analysis of the relationship between multiple mating and embryo survivorship. For control females, both episodes of postmating selection, resulting from eggs per mate and offspring survivorship, are negative and oppose the strong positive selection on body length from mating success (Table 1). Interestingly, we saw no evidence for this trade-off in the EE2 tanks, where selection differentials for premating sexual selection as well as for both postmating episodes of selection were all positive, favoring larger females (Table 1), but these results also must be interpreted with caution as the 95% confidence intervals for the selection differentials for postmating episodes overlapped zero. Regardless, our results do clearly show that sexual selection on females was not dramatically reduced as a result of EE2 exposure in Gulf pipefish.

**Table 1:** Selection differentials broken down into three episodes of selection for the control and EE2 treatments. We calculated the absolute selection differential (*s*), in cm, and the standardized selection differential (*s*'), in units of phenotypic standard deviations, for each tank separately and calculated means and 95% confidence intervals (bracketed values below the mean) across tanks. Episodes of selection include mating success, number of eggs transferred per mate, and embryo survivorship

Selection episode	Male *s* [95% CI]	%	Male *s*' [95% CI]	%	Female *s* [95% CI]	%	Female *s*' [95% CI]	%
Control								
Premating selection (mating success)	0.034 [−0.012, 0.079]	41.9	0.087 [−0.046, 0.220]	53.5	0.140 [−0.015, 0.295]	143.5	0.209 [−0.011, 0.429]	155.1
Postmating selection (eggs per mate)	0.037 [−0.001, 0.075]	46.0	0.051 [0.006, 0.096]	31.4	−0.026 [−0.093, 0.041]	−26.9	−0.044 [−0.174, 0.086]	−32.8
Postmating selection (embryo survivorship)	0.010 [−0.044, 0.064]	12.1	0.024 [−0.053, 0.102]	15.0	−0.016 [−0.029, −0.004]	−16.6	−0.030 [−0.056, −0.005]	−22.4
Total selection differential	0.080 [−0.002, 0.163]	100	0.162 [0.004, 0.321]	100	0.097 [−0.046, 0.241]	100	0.135 [−0.072, 0.341]	100
EE2								
Premating selection (mating success)	0.037 [−0.006, 0.081]	26.8	0.082 [−0.053, 0.216]	38.0	0.120 [0.029, 0.211]	77.4	0.236 [0.037, 0.436]	86.1
Postmating selection (eggs per mate)	0.100 [−0.016, 0.215]	71.4	0.133 [0.005, 0.261]	62.0	0.029 [−0.022, 0.080]	18.7	0.030 [−0.050, 0.109]	10.8
Postmating selection (embryo survivorship)	0.002 [−0.015, 0.019]	1.8	0.0 [−0.019, 0.019]	0.0	0.006 [−0.009, 0.021]	3.9	0.008 [−0.014, 0.031]	3.1
Total selection differential	0.139 [0.013, 0.266]	100	0.215 [0.067, 0.363]	100	0.155 [0.037, 0.272]	100	0.274 [0.050, 0.499]	100

Patterns of selection in males differed considerably from those in females. For instance, no single episode of selection made up the vast majority of the male selection differentials (Table 1). For both control and EE2 males, we see more of a balance between the contributions of the first two episodes. The second episode of selection measures reproductive success per pregnancy and suggests that larger males are receiving a greater number of eggs. This episode is statistically significant in control and EE2 treatments for the standardized selection (*s*') differential on male standard length and is probably best described as fecundity selection, because larger males, which typically have larger brood pouches, receive more eggs per mating (control: *n *=* *49, *r *=* *0.38, *P *=* *0.007; EE2: *n *=* *50, *r *=* *0.29, *P *=* *0.044).

We also calculated the total opportunity for selection and found that the decomposed episodes of *I* paralleled the results from our selection differentials (Table 2). The total opportunity for selection, as well as *I* for each of the three individual episodes, did not differ across the treatments for either sex. However, similar to our total selection differentials, we see that in both the control and EE2 treatments, the total opportunity for selection is statistically greater in females than that in males as evidenced by nonoverlapping 95% confidence intervals (Table 2). When we break down the sources of variation responsible for *I* into episodes, there are very similar patterns across each treatment within the sexes when compared to our decomposed selection differentials. Similar to *s* and *s*', we find that the first episode of selection, resulting from variance in mating success, is responsible for the majority of variance in female fitness and is statistically significant in both the control and EE2 replicates. On the other hand, the second and third episodes of selection only represent 4–4.6% and 0.6–0.9%, respectively, of the total opportunity for selection in females across treatments (Table 2). The total opportunity for selection in males was significantly lower than that in females, and the decomposition in males reveals different contributions of the various episodes of selection. In general, we found similar patterns in control and EE2 treatments for males. In particular, mating success (*I*_1_) and number of eggs per mate (*I*_2_) make the largest contribution to variation in fitness for males. Embryo survivorship (*I*_3_) made a small (i.e., 4–17.5%) but statistically significant contribution in both treatments (Table 2).

**Table 2:** Decomposition of the opportunity for selection (*I*) by selection episode. The selection episodes include number of mates, number of eggs transferred per mate, and offspring survivorship during the pregnancy. The decomposition of *I* follows Arnold and Wade (1984a,b), and the covariance terms are shown for completeness. See Arnold and Wade (1984a,b) for a more complete discussion of the interpretation of the various terms. For our purposes, the most important terms are *I*_1_, *I*_2_, and *I*_3_, which indicate the variance in relative fitness arising from our three episodes of selection. We conducted this partitioning for each tank separately and calculated means across tanks. We also report 95% confidence intervals (shown in brackets) across tanks

		Control tanks				EE2 tanks			
Source of variance in fitness	Symbol	Male value [95% CI]	Male%	Female value [95% CI]	Female%	Male value [95% CI]	Male%	Female value [95% CI]	Female%
Precopulatory sexual selection (mating success, *w*_1_)	*I*_1_	0.149 [−0.037, 0.335]	43.2	1.582 [0.878, 2.287]	96.4	0.132 [−0.001, 0.266]	34.0	1.204 [0.631, 1.778]	75.8
Postcopulatory selection arising from number of eggs transferred (eggs per mate, *w*_2_)	*I*_2_	0.098 [0.049, 0.147]	28.4	0.065 [0.022, 0.108]	4.0	0.194 [0.058, 0.329]	49.8	0.074 [0.028, 0.119]	4.6
Covariance between *w*_1_ and *w*_2_:									
Unweighted	CO*I*(1,2)	0.105 [−0.013, 0.224]	30.5	0.519 [0.383, 0.655]	31.6	0.102 [0.001, 0.203]	26.2	0.471 [0.328, 0.614]	29.6
Weighted by number of mates	CO*I*(1,2|1)	0.000 [0.000, 0.000]	0	−0.064 [−0.157, 0.028]	−3.9	0.000 [0.000, 0.000]	0	0.073 [−0.045, 0.192]	4.6
Change in covariance between number of eggs (*w*_1_*w*_2_) and eggs per mate (*w*_2_) caused by precopulatory sexual selection	CO*I*(12,2|1) – CO*I*(12,2)	−0.097 [−0.210, 0.017]	−28.0	−0.528 [−0.651, −0.404]	−32.2	−0.094 [−0.187, −0.001]	−24.2	−0.358 [−0.507, −0.210]	−22.5
Variance in number of eggs (*w*_1_*w*_2_)	Subtotal: *I*_12_	0.256 [0.072, 0.439]	74.1	1.574 [0.806, 2.341]	95.9	0.334 [0.207, 0.461]	85.8	1.464 [0.764, 2.165]	92.2
Postcopulatory selection, embryo survivorship (embryo success, *w*_3_)	*I*_3_	0.060 [0.004, 0.117]	17.5	0.009 [0.002, 0.016]	0.6	0.016 [0.004, 0.028]	4.0	0.014 [0.002, 0.026]	0.9
Covariance between number of eggs (*w*_1_*w*_2_) and embryo success (*w*_3_):									
Unweighted	CO*I*(12,3)	0.122 [0.000, 0.244]	35.3	0.540 [0.406, 0.674]	32.9	0.150 [0.046, 0.254]	38.7	0.481 [0.334, 0.627]	30.2
Weighted by number of eggs	CO*I*(12,3|2)	0.016 [−0.001, 0.033]	4.6	0.023 [−0.032, 0.077]	1.4	0.021 [0.000, 0.043]	5.4	0.055 [−0.009, 0.119]	3.4
Change in covariance between total fitness (*w*_1_*w*_2_*w*_3_) and embryo success (*w*_3_) caused by first two episodes of selection	CO*I*(123,3|2) – CO*I*(123,3)	−0.109 [−0.218, 0.000]	−31.6	−0.505 [−0.586, −0.424]	−30.8	−0.132 [−0.230, −0.035]	−34.0	−0.425 [−0.555, −0.295]	−26.7
Total opportunity for sexual selection (*w*_1_*w*_2_*w*_3_)	*I*	0.345 [0.154, 0.537]	100	1.641 [0.747, 2.535]	100	0.389 [0.228, 0.549]	100	1.589 [0.807, 2.371]	100

**Figure 3:**
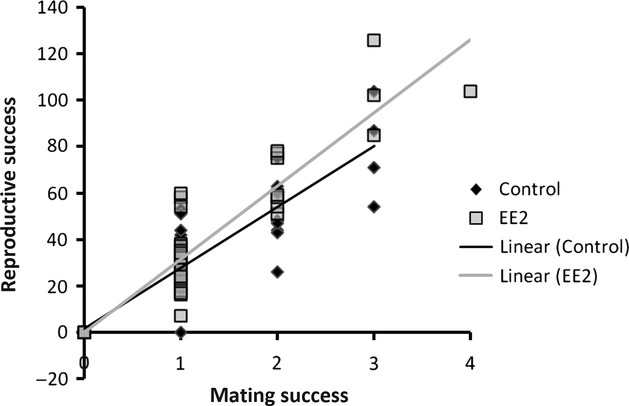
Absolute Bateman gradients for females in the EE2 and control treatments. The black line represents the linear regression of total number of offspring on number of mates for the control treatment, whereas the gray line shows the same linear regression for the EE2 treatment. The Bateman gradient for females in the EE2 treatment is significantly steeper than the Bateman gradient for females in the control replicates (ancova, with replicate as a random effect: *P = *0.009).

Our final metric related to sexual selection was the Bateman gradient, which we calculated for females and compared across treatments (Fig. 3). Our results show that the Bateman gradient for females is significantly steeper in the EE2 treatment than it is in the control treatment (ancova with replicate as a random effect*: P = *0.009), implying that sexual selection may be slightly stronger in the EE2 treatment than in the control treatment.

## Discussion

Exposure to EE2 has previously been shown to alter the mating behaviors of Gulf pipefish in binary choice tests, which suggested that EE2 could potentially disrupt the mating system and alter sexual selection. While we did find several changes in the mating system of Gulf pipefish as a result of low levels of EE2 exposure, we did not see a significant change in the strength of selection for either sex. The presence of 2 ng/L of EE2 in our experimental treatments did not hinder the ability of males to become pregnant. However, exposure to higher concentrations of EE2, including 5 ng/L in our pilot study and 100 ng/L in previous studies, even for a short period of time, significantly impacted male reproductive potential (Partridge et al. 2010). While EE2 concentrations of 5 ng/L are typically considered to be on the lower end of the range of EE2 levels detected in the natural environment, studies have shown that chronic exposure to these relatively lower levels of EE2 can sterilize male fishes (Kolpin et al. 2002; Kidd et al. 2007). Thus, high levels of EE2 could certainly result in population collapse due to a complete loss of male brooding in pipefish. However, under the lowest levels of EE2 exposure investigated in the present study, males were able to become pregnant and the nature of sexual selection acting on females remained mostly unchanged relative to control populations.

The most important observation in our study indicated that EE2 exposure affected the reproductive success of females, but the proximate effect appeared to be positive rather than the negative effect we would have predicted *a priori*. Females in the control group that mated multiply transferred fewer eggs per mate than females with only one mate, indicating that females may have rationed their eggs or become egg limited as they mated with additional males. In the case of EE2-exposed females, however, we found a different pattern: multiply mated females transferred a comparable number of eggs per mate compared with singly mated females. When we look at the total number of eggs produced by females, we see that females exposed to EE2 that mated multiply produced many more eggs overall than the multiply mated females in the control group. The multiply mated females did not differ in body length between the treatments, so this difference cannot be attributed simply to larger females having higher fecundity, a trend typically seen in other species of pipefish (Braga Goncalves et al. 2011). The most likely explanation for this observation is that exposure to low levels of EE2 stimulates egg production in female Gulf pipefish thereby increasing their reproductive rates and that females can only realize these enhanced reproductive rates by mating with multiple males. This interpretation is consistent with observations involving fathead minnows, in which short periods of EE2 exposure have been shown to increase egg production (Jobling et al. 2003). However, chronic low levels of exposure eventually cause decreased egg production in fathead minnows (Jobling et al. 2003). Several studies have also shown reproductive failure in zebrafish as a result of either long-term exposure to low levels of EE2 or brief exposure to higher levels (Van den Belt et al. 2003; Nash et al. 2004; Xu et al. 2008), raising the possibility that the short-term benefit that we observed in pipefish could be offset by reduced lifetime fitness for EE2-exposed females. Future studies involving a longer time frame will be necessary to address this question.

This increase in reproductive rates in EE2-exposed females also appeared to have an effect on the trade-off between pre- and postmating episodes of selection. In the control group, females that mated multiply were able to transfer more eggs than singly mated females over the entire experiment but in turn had more of their eggs fail to develop. This trade-off between greater mating success in the premating phase of selection and a decrease in offspring survivorship, a postmating mechanism, was not present in the EE2 multiply mated females. This observation raises the possibility that EE2-exposed females produce eggs of higher quality as a result of the excess estrogen resource. It is also important to note that while we did not dissect out the ovaries of unmated females, there did not visually appear to be any degradation of functioning ovaries in the females that did not mate.

As noted above, while an exposure of 2 ng/L seems to benefit females in the short term, EE2 exposure at 5 ng/L, a concentration on the lower side of the range of EE2 detected in the natural environment, results in devastating reproductive impacts on Gulf pipefish populations. Females are largely unaffected, but males are seriously compromised by this level of EE2 contamination. In our pilot study, males exposed to 5 ng/L failed to carry pregnancies to term, were unable to mate, and showed abnormal brood pouch morphology. At higher levels of exposure, the effects are even more dramatic. For instance, male Gulf pipefish exposed to 100 ng/L displayed secondary sexual traits that are normally only found in females, including iridescent bands and a deeply keeled abdomen. Furthermore, these males showed a reduced ability to mate even after being removed from the short-term EE2 exposure, with a minimum lag time of 4 days until pregnancy (Partridge et al. 2010). Thus, we predict that exposure in natural populations approaching 5 ng/L or higher will reduce the reproductive potential of Gulf pipefish populations and that the negative effects will be mediated almost entirely by the impact of EE2 exposure on males rather than on females. Several studies have documented levels of EE2 ranging from 5 ng/L to below, indicating that both of the concentrations in our study are within the range of possible EE2 contamination occurring in the environment where natural populations of the Gulf pipefish reside.

We did not see a breakdown in selection in the Gulf pipefish's sex-role-reversed mating system in populations exposed to EE2. Instead, we see no significant changes in the opportunity for selection in both male and female Gulf pipefish. As a result of increased female reproductive rates in EE2, we actually see a small but nonsignificant increase in selection acting on body length in both males and females. In a previous study by Saaristo et al. (2009b) documenting a breakdown in sexual selection in sand gobies, a species with strong sexual selection on males, sexual selection was disrupted due to the feminization of males. If the male traits that sand goby females use to choose their mates, such as aggressiveness and courtship displays, are disrupted by EE2 exposure, then sexual selection would be expected to break down. In our sex-role-reversed system, we did not detect a breakdown of selection as a result of male feminization. If anything, selection on females was slightly stronger in the EE2 treatment than in the control (as evidenced by the significantly steeper Bateman gradient for EE2-exposed females), which could be due to the greater number of eggs available and their increased survivorship, possibly causing an increase in female–female competition. Sand gobies have conventional sex roles with male–male competition for access to females, so it makes sense that feminization of males would reduce their ability to compete for mates. Part of the reason we found a different effect of EE2, compared with the complete collapse of selection acting on male sand gobies, certainly stems from the sex-role-reversed mating system of Gulf pipefish. Increased feminization of females appears to have made them more fecund and more able to compete for mates. However, feminization of male Gulf pipefish did not seem to affect the mating system until the males began to lose their ability to maintain functional brood pouches, a situation that results in a complete cessation of reproduction rather than quantitative changes in the intensity of sexual selection. It is also important to note that in the study conducted by Saaristo et al. (2009b), female sand gobies were not exposed to EE2. However, in the present study, we exposed both sexes to EE2 to investigate the effects of exposure in a setting that would more closely mimic contamination of the natural environment.

In summary, low levels of EE2 exposure enhanced reproduction in female Gulf pipefish and increased the fitness of multiply mated females during both pre- and postmating episodes of selection. EE2 exposure did not disrupt premating sexual selection in the Gulf pipefish mating system; if anything, sexual selection was slightly stronger in the exposed populations, owing to the increased fecundity of exposed females. Even though this study documented the effects of low levels of EE2 exposure on the Gulf pipefish mating system, it is critical to put the level of exposure in perspective. In our study, EE2 had a positive effect on female egg production at low levels of 2 ng/L. However, at EE2 exposure levels of 5 ng/L and higher, male receptivity decreased and male fitness plummeted to zero. In conclusion, this study has added to our knowledge on the effects of EE2 on pipefish, providing more insight into the effects EE2 has at the population level rather than on the individual level. Now that we have established that successful matings can occur under low levels of EE2 exposure, the next question is how these pollutants affect these fish at various stages of their life cycle, especially in terms of juvenile recruitment and population viability in EE2-contaminated waters.

## Data archiving statement

Data for this study are available at Dryad, doi:10.5061/dryad.vj5np.
